# Contrastive learning and mixture of experts enables precise vector embeddings in biological databases

**DOI:** 10.1038/s41598-025-98185-8

**Published:** 2025-04-29

**Authors:** Logan Hallee, Rohan Kapur, Arjun Patel, Jason P. Gleghorn, Bohdan B. Khomtchouk

**Affiliations:** 1 Center for Bioinformatics and Computational Biology, University of Delaware, Newark, USA; 2 Lincoln Laboratory, Massachusetts Institute of Technology, Boston, USA; 3 The College of the University of Chicago, Chicago, USA; 4 Department of Biomedical Engineering, University of Delaware, Newark, USA; 5 Department of Biomedical Engineering and Informatics, Luddy School of Informatics, Computing, and Engineering, Indiana University, Indianapolis, USA

**Keywords:** Natural language processing, Biomedical literature, Biological databases, Machine learning, Computational science, Information technology, Scientific data, Medical research

## Abstract

The advancement of transformer neural networks has significantly enhanced the performance of sentence similarity models. However, these models often struggle with highly discriminative tasks and generate sub-optimal representations of complex documents such as peer-reviewed scientific literature. With the increased reliance on retrieval augmentation and search, representing structurally and thematically-varied research documents as concise and descriptive vectors is crucial. This study improves upon the vector embeddings of scientific text by assembling domain-specific datasets using co-citations as a similarity metric, focusing on biomedical domains. We introduce a novel Mixture of Experts (MoE) extension pipeline applied to pretrained BERT models, where every multi-layer perceptron section is copied into distinct experts. Our MoE variants are trained to classify whether two publications are cited together (co-cited) in a third paper based on their scientific abstracts across multiple biological domains. Notably, because of our unique routing scheme based on special tokens, the throughput of our extended MoE system is exactly the same as regular transformers. This holds promise for versatile and efficient One-Size-Fits-All transformer networks for encoding heterogeneous biomedical inputs. Our methodology marks advancements in representation learning and holds promise for enhancing vector database search and compilation.

## Introduction

The remarkable success of transformer-based large language models (LLMs)^1^ has significantly increased our confidence in their abilities and outputs. Nowadays, LLMs are treated as *de facto* knowledge bases and have been adopted on a mass scale with the release of services like ChatGPT and open-source counterparts like Llama, Mistral, and DeepSeek-V3^2–4^. However, despite their widespread use, challenges persist, particularly concerning the accuracy and reliability of these models. For example, common issues like LLM hallucinations^5,6^ highlight the ongoing need for improvement. The ability to generate reliable vector embeddings and perform precise classification is crucial, especially for technologies that rely on information retrieval and web search.

One approach to further curate transformer latent spaces is to utilize contrastive learning to create sentence similarity models, initially revolutionizing sentiment analysis with broader applications in vector search^7–9^. More recently, the E5 line of models has demonstrated strong performance by applying contrastive learning on mean-pooled embeddings derived from the CCPairs dataset^10^. This resulted in a strong sentence similarity model that still has the top spot on the Massive Text Embedding Benchmark (MTEB) leaderboard at the time of writing^10,11^. However, as we showcase below, even strong sentence similarity models like E5 miss out-of-distribution domain-specific nuances^12,13^, resulting in sub-optimal representations of many important documents, including scientific literature.

Fortunately, several advancements have paved the way toward effective sentence similarity models over an arbitrary number of domains. Work from the metascience community has introduced co-citation networks as a practical way to gather many similar papers^14–22^. While this degree of similarity may not be perfect, co-citations have been shown to imply a high degree of similarity between papers^21^. Another promising advancement comes from the deep learning community with Mixture of Experts (MoE) models. Their learned input-dependent routing of information constitutes a promising multidomain / multitask learning architecture without significant added overhead^23^. Taking advantage of these methods, we propose the following MoE extension framework to build discriminative vector representations of input documents across diverse domains:

1. *Domain-specific fine-tuning* Apply contrastive fine-tuning methods to pretrained BERT (Bidirectional Encoder Representation Transformers) models using a predefined similarity heuristic, tailoring them to learn and understand domain-specific nuance.
2. *Universal applicability through mixture of experts (MoE)* Introduce a scalable method of seeding MoE models from dense pretrained transformers, aiming for a versatile “One-Size-Fits-All” model.

In this study, we conduct a case analysis on biomedical scientific literature - building a strong sentence similarity model that leverages co-citations as a similarity heuristic to differentiate niche literature across diverse domains from their textual abstracts alone. Our results show that the MoE extension framework improves LLMs performance in identifying semantically similar or niche intradisciplinary texts, showcasing a scalable method to produce effective vector representations that generalize across a wide range of scientific literature. Our methods substantially outperform general pretrained models and fine-tuned sentence similarity models, including science-oriented BERT models and Llama3.

## Methods

### Data compilation

We used co-citations as a similarity heuristic to generate sufficiently large training datasets for contrastive learning over scientific domains. Co-citations represent instances where two papers are cited together in a third paper. Our strategy enabled the production of large training datasets from small amounts of data due to the nonlinear scaling of citation graphs, as a single paper citing *N* other papers produces $N\atopwithdelims ()2$ co-citation pairs. For context, a dataset of 10,000 individual papers can produce well over 125,000 co-citation pairs. While this measurement of similarity is not perfect, co-citations have generally been shown to imply a high degree of similarity between papers^21^. We assume for our modeling purposes that two co-cited papers are more similar than two random papers, even if they are from the same field.

To build our dataset, we randomly chose five biomedical subfields with little overlap. The domains of choice include papers related to cardiovascular disease (CVD), chronic obstructive pulmonary disease (COPD), parasitic diseases, autoimmune diseases, and skin cancers. PubMed Central was queried with Medical Subject Heading (MeSH) terms for each domain, requiring at least one citation and an abstract present between 2010 and 2022. This means that within the time period, we kept the co-citation pairs of the possible $N\atopwithdelims ()2$ co-citations per paper that were returned from the same common MeSH terms. We sampled preferentially from samples co-cited more times when constructing our final dataset.

For evaluation, we constructed “negative” examples of abstract pairs that were not co-cited. The training dataset was split randomly in a 99:1 ratio followed by deduplication. We built negative pairs by pairing abstracts that had not been co-cited and had both been cited at least 15 times. This criteria allowed us to construct a representative evaluation set for binary classification with balanced classes, with 1’s for co-cited pairs and 0 if not. The exact dataset counts are outlined in Table 1.

**Table 1:** Training and evaluation set sizes across the biomedical domains used.

Domain	Abstracts	Sampled pairs	
		Training	Evaluation
COPD	6,379	132,453	2,676
CVD	13,328	181,000	4,584
skin cancer	5,268	85,805	1,734
parasitic	26,251	1,048,575	27,750
autoimmune	23,159	499,852	10,066
**Total**	**74,385**	**1,947,685**	**46,810**

### Transformer neural networks

The transformer architecture is adept at sequential processing and is state-of-the-art for various natural language processing (NLP) and vision tasks^24–30^. A transformer block comprised a self-attention layer and multi-layer perception (MLP) interleaved with skip connections. Full transformers were made of *T* transformer blocks stacked together^1^.

Prior to the transformer blocks is the token embedding process, where tokenization maps an input string into a list of *L* integers from a dictionary. These integers served as the indices for a matrix $W_e$, where each row is a learnable representative vector for that token, making $W_e\in \mathbb {R}^{v\times d}$ where *v* is the total number of unique tokens in the vocabulary and *d* an arbitrarily chosen hidden dimension. The initial embedding is $\mathbb {R}^{L\times d}$.

Each block in the transformer then transforms this embedding, i.e., the $i^{th}$ transformer block maps the embedding $X^{(i-1)} = [x_1^{(i-1)}, ..., x_L^{(i-1)}]^\top \in \mathbb {R}^{L\times d}$ to $X^{(i)} = [x_1^{(i)}, ..., x_L^{(i)}]^\top \in \mathbb {R}^{L \times d}$^1,31,32^. $X^{(T)}$ is the last hidden state of the network. The first part of this map is self-attention, which mixes information across the vectors, followed by the MLP which mixes information across *d*^31,33^.

Including the MLP, the entire transformer block can be written as:

$$\begin{aligned} X^{(i)} = \sigma (\text {Attention}(X^{(i-1)})W_1 + b_1)W_2 + b_2, \end{aligned}$$

where $b_1$ and $b_2$ are biases associated with learned linear transformations $W_1 \in \mathbb {R}^{d\times I}$ and $W_2 \in \mathbb {R}^{I\times d}$, where $I > d$. The activation function $\sigma$, e.g., ReLU or GeLU, introduces non-linearity^1^. More recently, biases are not included, which improves training stability, throughput, and final performance. Additionally, improvements like SwiGLU activation functions and rotary positional embeddings are also commonly utilized^3,4,34,35^.

GPT (Generative Pretrained Transformer) models, such as OpenAI’s GPT series (GPT-3, GPT-4, etc.), are designed for generative tasks and use transformer decoders^36–38^. They employ causal (unidirectional) attention, meaning each token attends only to previous tokens in the sequence, enabling autoregressive generation during inference. This allows them to predict the next word in a sequence without direct access to future words.

In contrast, BERT models utilize transformer encoders with bidirectional attention, meaning they can attend to all tokens within an input simultaneously. This structure enables them to capture additional contextual dependencies, making them well-suited for tasks like text classification and sentence similarity^39^. Unlike GPT models, BERT is trained using a masked language modeling (MLM) objective, where some tokens are randomly hidden, requiring the model to predict them based on the surrounding context.

#### Mixture of Experts

Mixture of Experts (MoE) models add a linear layer or router network to each transformer block, which outputs logits from $H^{(i)}$. These logits route $H^{(i)}$ to multiple equivalent copies of the MLP section with different weights called experts^40^. In many transformer variants, this routing is typically done on a per-token basis, allowing for experts to specify in language classes like punctuation, nouns, numbers, etc^41^. We chose sentence-wise routing of the entire $H^{(i)}$ so that we could purposely structure our experts for specific domains^42^.

**Fig. 1:**
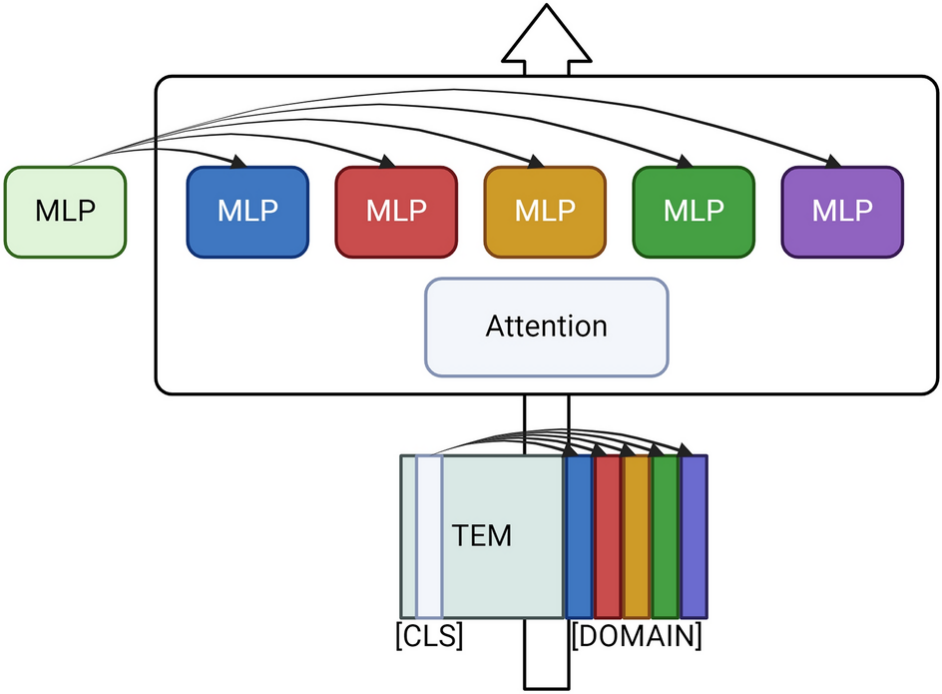
Visualization of our MoE extension pipeline, where the MLP of each transformer network is copied into equivalent experts to further differentiate during training. Additionally, domain-specific special tokens are seeded from the pretrained Token Embedding Matrix (TEM) using the [CLS] token, which is replaced with the correct domain token upon tokenization.

Controlling the routing of $H^{(i)}$, allowed for a one-size-fits-all approach to text classification where one expert per transformer layer was an expert in a specific domain. For faster fine-tuning, we utilized pretrained models for our novel MoE extension approach (Fig. 1). As such, each MLP section was copied into five identical components to be differentiated during training. We also removed the learned router entirely and routed examples to a specific expert based on which domain the text comes from. The final MoE models had five experts each, where all COPD inputs were routed to a single expert and all CVD inputs to another, etc. To further enhance the nuance behind the representations built from our model, and to allow for the attention layers to distinguish which type of input was fed to the model, we added special tokens for each domain, e.g., [CVD], [COPD], etc. The token embedding for these new special tokens was seeded with the pretrained weight from the [CLS] token, and the [CLS] token was replaced with the correct domain token during tokenization. As such, the domain tokens were equivalent to the [CLS] token before further training.

#### Models of choice

We chose the recent ModernBERT base model as the pretrained model of choice for our experiments^35^. This bidirectional model employs efficient implementations of masking and attention to speed up training and inference while reducing memory costs. Local attention was used in most layers, with global attention at every third layer. We trained ModernBERT directly without modification and with MoE extension. The ModernBERT models offer much higher NLP benchmark performance per parameter than the first generation of BERT models following the 2019 release and subsequent fine-tuning releases^35^.

To benchmark against our training scheme, we compiled several popular BERT-like models, BERT models fine-tuned on scientific literature, sentence similarity models, and a recent SOTA GPT-like transformer (Table 2). ModernBERT, BERT, and RoBERTa have all been solely pretrained with MLM objectives^35,39,43^. SciBERT, BioBERT, and PubmedBERT have all been trained further on a scientific corpus with additional MLM^44–46^. all-MiniLM-L6-v2 (Mini), MPNet, and E5 have been fine-tuned using contrastive learning for sentence similarity and embedding-based tasks^10,47–53^. Llama-3.2 is a state-of-the-art “small” generative language model that has seen wide use and success in local and open-source use cases^3^. We also benchmarked against a basic term frequency-inverse document frequency (TF-IDF), acting as a baseline for expected performance^54^.

All transformer models were downloaded and used with the Huggingface [SPSVERBc1SPS] package, leveraging custom embedding classes for efficient resource management. The TF-IDF scheme was fit on each domain separately using Python’s scikit-learn implementation with 4,096 maximum features^54,55^. The wide diversity in representation learning models allowed for an effective comparison to our training scheme.

**Table 2:** Summary of the models used in the study.

Model	Parameter count (millions)	Huggingface path
Llama-3.2	1236	meta-llama/Llama-3.2-1B
ModernBERT$_{large}$	395	answerdotai/ModernBERT$_{large}$
BERT$_{large}$	336	google-bert/bert$_{large}$-uncased
E5$_{large}$	335	intfloat/e5$_{large}$-v2
RoBERTa$_{large}$	335	FacebookAI/roberta$_{large}$
MoE$_{all}$ (ours)	150 active, 384 total	GleghornLab/MoE$_{all}$-sentence
ModernBERT$_{base}$	149	answerdotai/ModernBERT$_{base}$
Roberta$_{base}$	125	FacebookAI/roberta$_{base}$
SciBERT	110	allenai/scibert_scivocab_uncased
BERT$_{base}$	110	google-bert/bert$_{base}$-uncased
E5$_{base}$	109	intfloat/e5$_{base}$-v2
PubmedBERT	109	microsoft/BiomedNLP-BiomedBERT$_{base}$-uncased-abstract-fulltext
MPNet	109	sentence-transformers/all-mpnet$_{base}$-v2
BioBERT	108	dmis-lab/biobert-v1.1
Mini	23	sentence-transformers/all-MiniLM-L6-v2

### Training strategy

To minimize training and inference time, we chose to use abstracts rather than entire papers as the text input to the model. Abstracts represent a human-generated summarized version of a paper and, as a result, include much of the relevant textual information contained in a paper. We trained regular ModernBERT$_{base}$ models (single expert or SE models) on one domain at a time, on every domain (SE$_{all}$), and our MoE extended model (MoE$_{all}$) on every domain.

The training objective was to summarize two paired mini-batches of abstracts separately. The abstract of index *i* in each batch was a co-cited abstract pair. The last hidden state of the model $H^{(L)}$ was mean pooled to build fixed-length vector representations of each batch. Then, we compared these embeddings with the variant of the Multiple Negative Rankings (MNR) loss used to train cdsBERT^56,57^. MNR Loss is a loss function that has seen significant success with sentence embedding problems^58^ and was highly successful in our local experiments. Our variant used dot products as an inter-batch similarity heuristic and constructed the targets based on the average intra-batch dot products. The loss was formulated as follows:

$$\begin{aligned} \tilde{L}(A, B)&= \sum _{i=1}^b H(\text {argmax}_{j=1,...,b}(A_{j,:}^\intercal A_{i,:} + B_{j,:}^\intercal B_{i,:}) \\ L(B_1, B_2)&= \tilde{L}(B_1, B_2) + \tilde{L}(B_2, B_1), \end{aligned}$$

where *b* is the batch size, $B_i \in \mathbb {R}^{b \times d}$ is mini-batch *i* and *H* is the cross-entropy. Batches $B_1, B_2$ must be paired such that element $i=i$ are “similar,” in our case co-cited, and assumed to be dissimilar for other indices $i \ne j$ of a paired batch. This can be easily achieved by passing two paired batches to the model in two forward passes and combining their gradients for one backward pass in a standard autograd library. We used PyTorch for our experiments^59^.

The advantage of MNR losses and their variants is precisely this property requiring only positive/similar text pairs, generating negative/dissimilar text pairs from the other indices $i \ne j$ of the mini-batch. As a result, MNR loss removed the need to generate dissimilar text pairs for our training dataset under the assumption that the random chance of finding a similar paper randomly that is co-cited, is sufficiently small. Indices $i \ne j$ during single-domain training would be randomly paired papers from the same field, while during multi-domain training, it could be two random papers from different fields or the same. In either case, this approach satisfied our modeling assumptions that two co-cited papers were more similar than two random papers.

During training, we randomly switched the order of the two input abstract pairs to prevent any bias in how they were fed to the loss function. A batch size $b=16$ was chosen for computational throughput and minimizing the chance for multiple positive abstract pairs showing up in a mini-batch. We trained models with a cosine learning rate scheduler with warm up using a learning rate of $1e^{-4}$, and performed periodic validation to measure training progress. Training was halted when a patience of 5 was exceeded for the evaluation set $F1_{max}$.

### Evaluation strategy

**Fig. 2:**
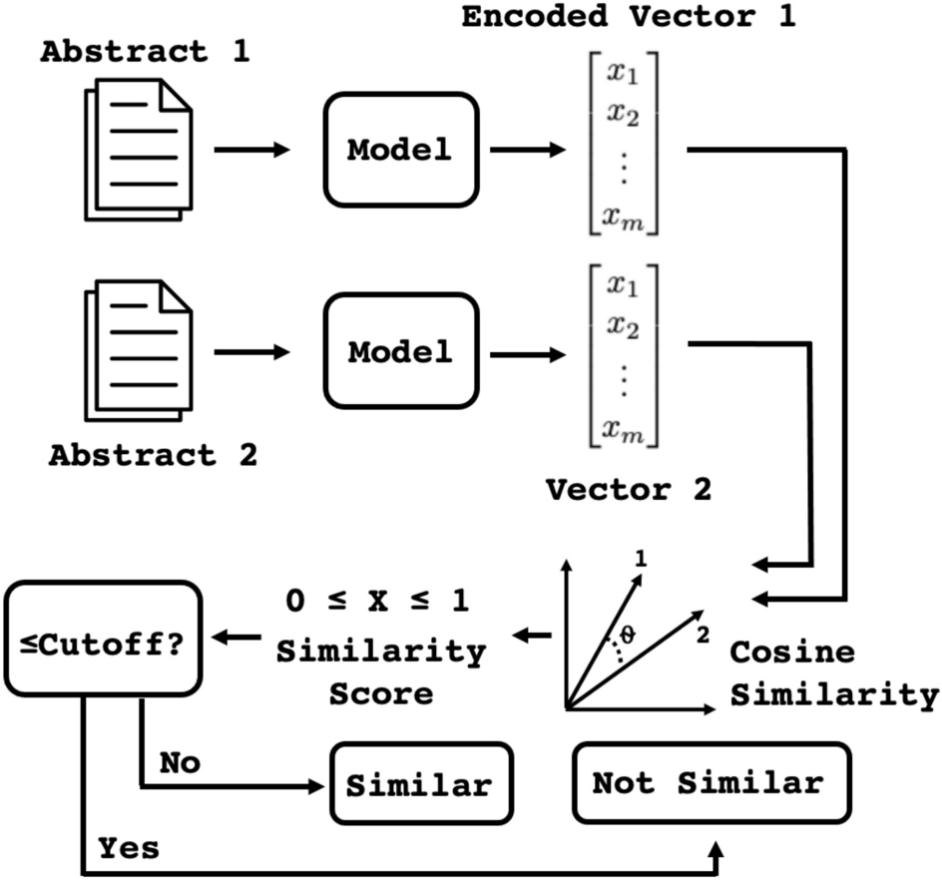
Method for determination of abstract pair similarity for model evaluation.

All models were evaluated on the evaluation sets separately for each domain, as well as averaged together. We used cosine similarity between two vectors extracted from an abstract pair to classify the abstracts as co-cited (similar) or not, given a threshold, shown in Fig. 2. Cosine similarity is a common vector similarity measure ranging from -1 to 1, where -1 is exactly the opposite and 1 occurs for a pair of the same vector. We thresholded the cosine similarly to create a decision boundary and measured the $F1_{max}$ to evaluate performance^60,61^. $F1_{max}$ is the maximum F1 score calculated for all possible thresholds for a reported metric, calculated using a precision-recall curve. While typically used for imbalanced multilabel classification, randomly choosing a binary threshold for the reported F1 would not be a fair comparison of different models. For example, one model may perform much better with a cosine similarity threshold at 0.5 for abstract text similarity compared to 0.4, or vice versa. We also reported average precision and recall at the optimal threshold found for $F1_{max}$, the ROC-AUC, as well as the average cosine similarity ratio between positive examples and negative examples. This ratio showcases the average discriminative power of a model, where a higher ratio implies that positive and negative examples are more separable.

## Results

The results for all domains averaged together are shown in Table 3. On average, our full approach with MoE extension and contrastive learning (MoE$_{all}$), showcased the highest performance with an $F1_{max}$ of 0.8875 across all domains. For single domain evaluation, we expected single expert (SE) models that were fine-tuned for only that domain, for example, SE$_{cancer}$ for the parasitic diseases, to perform the best. For skin cancer (Table 5), COPD (Table 6), CVD (Table 7), and parasitic (Table 8) literature this was the case. This trend did not hold in the autoimmune domain (Table 4), with MoE$_{all}$ narrowly outperforming SE$_{autoimmune}$. Of note, MoE$_{all}$ and SE$_{all}$ failed to perform better than base sentence similarity models on the CVD abstracts, where only SE$_{cvd}$ outperformed MPnet, E5, and Mini. Statistical analysis of all pairwise model performance can be found in the supplemental materials (Supplemental Fig. 1–6).

**Table 3:** Metrics for binary prediction of co-citation between two input abstracts via cosine similarity averaged across all evaluation sets, sorted by $F1_{max}$. Threshold refers to the optimal decision cutoff using the cosine similarities of that dataset. SE models use their domain token for all domains. Models trained in this work are highlighted in bold.

Model	F1	Precision	Recall	Threshold	Ratio	ROC-AUC
**MoE**$_{{{\varvec{all}}}}$	**0.8875**	**0.8610**	**0.9166**	**0.7083**	**1.7189**	**0.9426**
**SE**$_{{{\varvec{all}}}}$	**0.8770**	**0.8510**	**0.9067**	**0.7475**	**1.5535**	**0.9338**
**SE**$_{{{\varvec{cancer}}}}$	**0.8311**	**0.7809**	**0.9055**	**0.6552**	**1.7491**	**0.8606**
MPNet	0.8038	0.7535	0.8611	0.4364	1.7541	0.8762
Mini	0.7940	0.7351	0.8631	0.3822	1.7706	0.8641
E5$_{base}$	0.7910	0.7322	0.8601	0.8082	1.0676	0.8664
E5$_{large}$	0.7908	0.7323	0.8594	0.8020	1.0664	0.8671
**SE**$_{{{\varvec{autoimmune}}}}$	**0.7702**	**0.7042**	**0.8626**	**0.7258**	**1.3705**	**0.8151**
TF-IDF	0.7523	0.7024	0.8097	0.0744	2.2966	0.8209
Llama-3.2-1B	0.7489	0.6894	0.8197	0.8403	1.0769	0.8174
**SE**$_{{{\varvec{cvd}}}}$	**0.7458**	**0.6596**	**0.8637**	**0.6669**	**1.3775**	**0.7947**
**SE**$_{{{\varvec{copd}}}}$	**0.7347**	**0.6441**	**0.8689**	**0.7264**	**1.1872**	**0.7715**
**SE**$_{{{\varvec{cancer}}}}$	**0.7132**	**0.6200**	**0.8526**	**0.5719**	**1.2614**	**0.7416**
BioBERT	0.7123	0.6314	0.8168	0.9384	1.0154	0.7646
PubmedBERT	0.7111	0.6488	0.7867	0.9853	1.0039	0.7614
RoBERTa$_{large}$	0.6999	0.5815	0.8789	0.9949	1.0011	0.7395
SciBERT	0.6992	0.6010	0.8360	0.8648	1.0311	0.7400
ModernBERT$_{large}$	0.6991	0.6014	0.8347	0.9350	1.0146	0.7378
BERT$_{large}$	0.6987	0.6069	0.8232	0.8857	1.0302	0.7370
BERT$_{base}$	0.6956	0.5816	0.8652	0.8417	1.0411	0.7296
ModernBERT$_{base}$	0.6919	0.5749	0.8687	0.9427	1.0120	0.7236
RoBERTa$_{base}$	0.6800	0.5487	0.8940	0.9834	1.0031	0.6998

**Table 4:** Metrics for binary prediction of co-citation between two input abstracts via cosine similarity for the autoimmune evaluation set, sorted by $F1_{max}$. Threshold refers to the optimal decision cutoff using the cosine similarities of that dataset. Models trained in this work are highlighted in bold.

Model	F1	Precision	Recall	Threshold	Ratio	ROC-AUC
**MoE**$_{{{\varvec{all}}}}$	**0.8908**	**0.8692**	**0.9136**	**0.7055**	**1.7709**	**0.9552**
**SE**$_{{{\varvec{autoimmune}}}}$	**0.8904**	**0.8659**	**0.9164**	**0.6205**	**2.1502**	**0.9541**
**SE**$_{{{\varvec{all}}}}$	**0.8845**	**0.8586**	**0.9120**	**0.7512**	**1.5411**	**0.9474**
MPNet	0.8198	0.7927	0.8488	0.3851	1.9632	0.8928
Mini	0.8182	0.8114	0.8252	0.3509	2.0583	0.8898
E5$_{large}$	0.8099	0.7817	0.8403	0.8005	1.0734	0.8859
E5$_{base}$	0.7999	0.7899	0.8103	0.8123	1.0710	0.8739
TF-IDF	0.7774	0.7440	0.8140	0.0709	2.6626	0.8454
**SE**$_{{{\varvec{cvd}}}}$	**0.7502**	**0.6577**	**0.8728**	**0.5747**	**1.4545**	**0.8131**
Llama-3.2-1B	0.7442	0.7068	0.7858	0.8337	1.0813	0.8136
**SE**$_{{{\varvec{copd}}}}$	**0.7329**	**0.6543**	**0.8329**	**0.6757**	**1.2351**	**0.7825**
BioBERT	0.7155	0.6282	0.8309	0.9347	1.0167	0.7645
PubmedBERT	0.7128	0.6429	0.7997	0.9842	1.0036	0.7618
SciBERT	0.7060	0.5996	0.8585	0.8511	1.0357	0.7484
RoBERTa$_{large}$	0.7034	0.5929	0.8645	0.9947	1.0013	0.7401
ModernBERT$_{large}$	0.6993	0.5868	0.8651	0.9310	1.0154	0.7361
**SE**$_{{{\varvec{cancer}}}}$	**0.6920**	**0.5639**	**0.8955**	**0.4233**	**1.2759**	**0.7218**
BERT$_{base}$	0.6917	0.5920	0.8319	0.8563	1.0385	0.7198
BERT$_{large}$	0.6914	0.5833	0.8486	0.8816	1.0292	0.7242
ModernBERT$_{base}$	0.6904	0.5894	0.8331	0.9428	1.0122	0.7165
**SE**$_{{{\varvec{cancer}}}}$	**0.6890**	**0.5866**	**0.8347**	**0.7283**	**1.1047**	**0.7137**
RoBERTa$_{base}$	0.6872	0.5582	0.8937	0.9830	1.0036	0.7089

**Table 5:** Metrics for binary prediction of co-citation between two input abstracts via cosine similarity for the skin cancer evaluation set, sorted by $F1_{max}$. Threshold refers to the optimal decision cutoff using the cosine similarities of that dataset. Models trained in this work are highlighted in bold.

Model	F1	Precision	Recall	Threshold	Ratio	ROC-AUC
**SE**$_{{{\varvec{cancer}}}}$	**0.8509**	**0.8308**	**0.8720**	**0.6680**	**1.4538**	**0.9203**
**MoE**$_{{{\varvec{all}}}}$	**0.7687**	**0.7130**	**0.8339**	**0.8505**	**1.1447**	**0.8226**
**SE**$_{{{\varvec{all}}}}$	**0.7301**	**0.6070**	**0.9158**	**0.7867**	**1.1066**	**0.7724**
Llama-3.2-1B	0.6856	0.5891	0.8201	0.8249	1.0442	0.6863
SciBERT	0.6793	0.5289	0.9493	0.8387	1.0176	0.6392
E5$_{large}$	0.6769	0.5455	0.8916	0.7891	1.0272	0.6890
E5$_{base}$	0.6759	0.5404	0.9020	0.7970	1.0270	0.6838
**SE**$_{{{\varvec{copd}}}}$	**0.6756**	**0.5291**	**0.9343**	**0.6925**	**1.0549**	**0.6214**
TF-IDF	0.6747	0.5284	0.9331	0.0437	1.5295	0.6752
RoBERTa$_{large}$	0.6747	0.5165	0.9723	0.9938	1.0006	0.6518
ModernBERT$_{large}$	0.6735	0.5130	0.9804	0.9086	1.0076	0.6162
BioBERT	0.6733	0.5245	0.9400	0.9268	1.0077	0.6399
BERT$_{large}$	0.6723	0.5291	0.9216	0.8729	1.0155	0.6300
PubmedBERT	0.6716	0.5189	0.9516	0.9799	1.0026	0.6289
MPNet	0.6711	0.5086	0.9862	0.2200	1.1740	0.6545
**SE**$_{{{\varvec{cvd}}}}$	**0.6701**	**0.5129**	**0.9666**	**0.6468**	**1.0530**	**0.6317**
BERT$_{base}$	0.6693	0.5122	0.9654	0.8139	1.0190	0.6223
Mini	0.6680	0.5147	0.9516	0.2560	1.1917	0.6682
RoBERTa$_{base}$	0.6680	0.5032	0.9931	0.9759	1.0012	0.5989
**SE**$_{{{\varvec{cancer}}}}$	**0.6677**	**0.5014**	**0.9988**	**0.4490**	**1.0502**	**0.6236**
ModernBERT$_{base}$	0.6677	0.5143	0.9516	0.9273	1.0062	0.6150
**SE**$_{{{\varvec{autoimmune}}}}$	**0.6669**	**0.5003**	**1.0000**	**0.4453**	**1.0659**	**0.6545**

**Table 6:** Metrics for binary prediction of co-citation between two input abstracts via cosine similarity for the COPD evaluation set, sorted by $F1_{max}$. Threshold refers to the optimal decision cutoff using the cosine similarities of that dataset. Models trained in this work are highlighted in bold.

Model	F1	Precision	Recall	Threshold	Ratio	ROC-AUC
**SE**$_{{{\varvec{copd}}}}$	**0.8270**	**0.8215**	**0.8326**	**0.6844**	**1.4667**	**0.9043**
**MoE**$_{{{\varvec{all}}}}$	**0.7861**	**0.7039**	**0.8901**	**0.7461**	**1.2528**	**0.8548**
**SE**$_{{{\varvec{all}}}}$	**0.7661**	**0.7207**	**0.8176**	**0.8515**	**1.1422**	**0.8293**
Llama-3.2-1B	0.6940	0.5965	0.8296	0.8434	1.0463	0.7272
**SE**$_{{{\varvec{cvd}}}}$	**0.6897**	**0.5828**	**0.8445**	**0.6226**	**1.1619**	**0.6922**
SciBERT	0.6870	0.5591	0.8909	0.8502	1.0264	0.6968
BioBERT	0.6868	0.5397	0.9439	0.9249	1.0112	0.7023
BERT$_{base}$	0.6856	0.5705	0.8587	0.8503	1.0306	0.7004
PubmedBERT	0.6854	0.5750	0.8483	0.9838	1.0029	0.7042
RoBERTa$_{large}$	0.6851	0.5816	0.8333	0.9949	1.0009	0.7044
BERT$_{large}$	0.6849	0.5676	0.8632	0.8822	1.0224	0.6970
ModernBERT$_{base}$	0.6810	0.5358	0.9342	0.9344	1.0100	0.6937
ModernBERT$_{large}$	0.6798	0.5289	0.9514	0.9226	1.0104	0.6850
E5$_{base}$	0.6796	0.5601	0.8640	0.8210	1.0273	0.7031
RoBERTa$_{base}$	0.6784	0.5412	0.9088	0.9830	1.0027	0.6749
E5$_{large}$	0.6771	0.5595	0.8572	0.8146	1.0275	0.7027
**SE**$_{{{\varvec{autoimmune}}}}$	**0.6742**	**0.5201**	**0.9581**	**0.6473**	**1.0717**	**0.6641**
MPNet	0.6728	0.5601	0.8423	0.4736	1.1831	0.7003
**SE**$_{{{\varvec{cancer}}}}$	**0.6716**	**0.5122**	**0.9753**	**0.6528**	**1.0415**	**0.6148**
TF-IDF	0.6696	0.5288	0.9126	0.0737	1.4375	0.6832
**SE**$_{{{\varvec{cancer}}}}$	**0.6696**	**0.5166**	**0.9514**	**0.5194**	**1.0755**	**0.6155**
Mini	0.6678	0.5023	0.9963	0.1744	1.1585	0.6733

**Table 7:** Metrics for binary prediction of co-citation between two input abstracts via cosine similarity for the CVD evaluation set, sorted by $F1_{max}$. Threshold refers to the optimal decision cutoff using the cosine similarities of that dataset. Models trained in this work are highlighted in bold.

Model	F1	Precision	Recall	Threshold	Ratio	ROC-AUC
**SE**$_{{{\varvec{cvd}}}}$	**0.9527**	**0.9353**	**0.9708**	**0.7217**	**2.4054**	**0.9876**
MPNet	0.9297	0.9241	0.9354	0.4246	2.7391	0.9754
E5$_{base}$	0.9257	0.9410	0.9110	0.8225	1.1112	0.9695
E5$_{large}$	0.9199	0.9284	0.9114	0.8157	1.1074	0.9671
Mini	0.9151	0.9084	0.9219	0.3782	2.8264	0.9697
**MoE**$_{{{\varvec{all}}}}$	**0.9060**	**0.8858**	**0.9271**	**0.8022**	**1.5423**	**0.9668**
**SE**$_{{{\varvec{all}}}}$	**0.8995**	**0.8916**	**0.9075**	**0.8253**	**1.4460**	**0.9640**
**SE**$_{{{\varvec{copd}}}}$	**0.8937**	**0.9093**	**0.8787**	**0.8286**	**1.4004**	**0.9538**
**SE**$_{{{\varvec{autoimmune}}}}$	**0.8794**	**0.8840**	**0.8748**	**0.8162**	**1.4353**	**0.9453**
TF-IDF	0.8778	0.8799	0.8757	0.1003	3.7699	0.9333
Llama-3.2-1B	0.8679	0.8365	0.9018	0.8477	1.1316	0.9385
**SE**$_{{{\varvec{cancer}}}}$	**0.8446**	**0.8302**	**0.8595**	**0.8330**	**1.2317**	**0.9115**
**SE**$_{{{\varvec{cancer}}}}$	**0.8258**	**0.8435**	**0.8089**	**0.7378**	**1.5186**	**0.8980**
SciBERT	0.7954	0.7529	0.8429	0.8670	1.0569	0.8626
PubmedBERT	0.7943	0.7811	0.8080	0.9854	1.0068	0.8561
BioBERT	0.7865	0.7649	0.8093	0.9389	1.0246	0.8599
RoBERTa$_{large}$	0.7844	0.7466	0.8264	0.9955	1.0020	0.8598
ModernBERT$_{large}$	0.7800	0.7287	0.8390	0.9410	1.0228	0.8422
ModernBERT$_{base}$	0.7641	0.7113	0.8255	0.9484	1.0211	0.8362
BERT$_{large}$	0.7526	0.6870	0.8320	0.8904	1.0454	0.8187
BERT$_{base}$	0.7428	0.6805	0.8176	0.8659	1.0592	0.8108
RoBERTa$_{base}$	0.7320	0.6805	0.7919	0.9870	1.0052	0.7970

**Table 8:** Metrics for binary prediction of co-citation between two input abstracts via cosine similarity for the parasitic evaluation set, sorted by $F1_{max}$. Threshold refers to the optimal decision cutoff using the cosine similarities of that dataset. Models trained in this work are highlighted in bold.

Model	F1	Precision	Recall	Threshold	Ratio	ROC-AUC
**SE**$_{{{\varvec{cancer}}}}$	**0.9060**	**0.8866**	**0.9262**	**0.6125**	**2.1802**	**0.9668**
**MoE**$_{{{\varvec{all}}}}$	**0.9004**	**0.8783**	**0.9237**	**0.6812**	**1.8100**	**0.9634**
**SE**$_{{{\varvec{all}}}}$	**0.8905**	**0.8694**	**0.9127**	**0.7208**	**1.6434**	**0.9567**
MPNet	0.8105	0.7698	0.8556	0.4600	1.7108	0.8829
Mini	0.8011	0.7480	0.8624	0.4044	1.7149	0.8722
E5$_{base}$	0.7933	0.7437	0.8499	0.8082	1.0659	0.8709
E5$_{large}$	0.7904	0.7381	0.8507	0.8021	1.0637	0.8691
Llama-3.2-1B	0.7460	0.6829	0.8218	0.8425	1.0717	0.8143
TF-IDF	0.7442	0.7048	0.7882	0.0744	2.1749	0.8158
**SE**$_{{{\varvec{autoimmune}}}}$	**0.7242**	**0.6464**	**0.8233**	**0.7742**	**1.1249**	**0.7769**
**SE**$_{{{\varvec{cvd}}}}$	**0.7202**	**0.6314**	**0.8381**	**0.6968**	**1.2209**	**0.7689**
BioBERT	0.7108	0.6159	0.8403	0.9384	1.0143	0.7648
PubmedBERT	0.7103	0.6362	0.8040	0.9855	1.0037	0.7606
**SE**$_{{{\varvec{copd}}}}$	**0.7039**	**0.5867**	**0.8797**	**0.7341**	**1.1159**	**0.7376**
BERT$_{large}$	0.6987	0.6117	0.8146	0.8858	1.0298	0.7393
**SE**$_{{{\varvec{cancer}}}}$	**0.6979**	**0.6002**	**0.8335**	**0.5975**	**1.2195**	**0.7297**
BERT$_{base}$	0.6964	0.5916	0.8462	0.8417	1.0415	0.7337
ModernBERT$_{large}$	0.6964	0.5974	0.8346	0.9350	1.0138	0.7328
RoBERTa$_{large}$	0.6929	0.5900	0.8395	0.9953	1.0010	0.7287
SciBERT	0.6922	0.5871	0.8430	0.8690	1.0267	0.7326
ModernBERT$_{base}$	0.6880	0.5724	0.8622	0.9446	1.0110	0.7167
RoBERTa$_{base}$	0.6742	0.5418	0.8924	0.9834	1.0028	0.6885

## Discussion

Our work advances the use of transformer language models with a focus on improving their domain-specific understanding and document-wide comprehension from summaries (abstracts). We have shown that popular pretrained models cannot distinguish the differences in highly discriminative scientific literature despite further fine-tuning for sentence similarity tasks with contrastive learning or additional MLM on scientific papers. For example, when examining the COPD domain results (Table 6), all evaluated pretrained models showcase random or near random performance, with no model achieving even a 0.7 $F1_{max}$. We assume this phenotype is even worse in prompting scenarios when considering common prompt construction and formatting. If a user were to ask ChatGPT if multiple scientific documents were similar or potentially co-cited, they would have to paste the documents together in the same input. When considering the self-attention mechanism, the semantic similarity of related tokens across the multiple documents may prevent effective distinction between the documents, as portions of each document will attend highly to each other even if they are “different” as defined by a desired discrimination. Therefore, a document-wise embedding approach is much more tractable, enforcing that multiple documents are input separately and embedded in a close vector space if similar enough.

As vector databases become more prevalent for search and retrieval tasks, the quality of these numerical representations becomes increasingly important. Our innovative framework, which incorporates contrastive learning through a custom MNR variant, novel special tokens, and MoE seeding, extension, and forced routing techniques, significantly enhances vector-based classification compared to pretrained transformers. We leveraged co-citation networks to construct large datasets of similar abstracts and applied our framework to scientific literature. This created nuanced representations with a specific focus on discriminative biomedical domains.

Specifically, MoE$_{all}$ and SE$_{all}$ performed the best on average, with 0.8875 and 0.8770 $F1_{max}$, respectively. Whereas SE models trained on a single domain were the best performers for that domain. This was the case for all domains except for autoimmune, where MoE$_{all}$ narrowly outperformed SE$_{autoimmune}$with a $F1_{max}$ of 0.8908 vs. 0.8904. MoE$_{all}$ routinely outperformed SE versions of the models with respect to the vector ratio metrics, often 1.7 vs. 1.5 between MoE$_{all}$ and SE$_{all}$, highlighting that the MoE extensions increased average separation of co-cited papers against others in the same field. Interestingly, the TF-IDF scheme often had the highest vector ratio, implying the highest average separation. Due to its subpar $F1_{max}$ and low threshold for binary classification, we can conclude that TF-IDF may be “over-confident” in general, placing small text motifs in a unique vector-space, perhaps missing the nuance that trained language models can capture.

Importantly, the SE$_{all}$ model with no MoE extension also performed exceedingly well, almost performing on par with MoE$_{all}$ in $F1_{max}$ and ratio. In our previous experiments and preprint, we used SciBERT for the classification of co-cited scientific documents^62^. With a SciBERT base model, the SE models trained on each domain outperformed the MoE version by a large margin, whereas the MoE version outperformed SE$_{all}$ by an even larger margin. We attribute this difference in performance to ModernBERT, a new optimized language model with excellent representations, as being a stronger base model for experimentation. As a result, the MoE extension had less of an effect than previous experiments using SciBERT.

Compellingly, when looking at the SE performance for models trained on one domain but evaluated on all domains (Table 3), they outperform many of the base models. For example, SE$_{cancer}$ outperforms even the sentence similarity models, which is not surprising considering that the parasitic data comprises a large portion of the overall dataset size. More interestingly, the SE$_{autoimmune}$ outperforms Llama-3.2, and SE$_{cvd}$, SE$_{copd}$, and SE$_{cancer}$ outperform all of the MLM-trained BERT models. This implies that when training on one scientific domain, performance is not significantly hampered across other unrelated domains. This is observed especially between the SE models and the weights they were seeded from, ModernBERT$_{base}$, with a 0.02 - 0.08 higher $F1_{max}$ and 0.2 - 0.4 increase in vector ratio.

Of course, our training scheme has limitations as well. For datasets that are already easily discriminated, such extensive fine-tuning may harm the overall performance. We support this notion when looking at the CVD domain results. The pretrained models had much higher natural scores, implying that these documents are already fairly separable, with a strong 2.7 ratio from MPnet, 2.8 ratio from Mini, and 3.8 ratio for TF-IDF. This led to MPnet, E5, and Mini outperforming MoE$_{all}$ and SE$_{all}$. It is also unclear if our scheme is resilient to datasets with vector ratios that the pretrained models disagree with. For example, even though we see low $F1_{max}$ across the base pretrained models, they all result in a ratio above 1.0 for every dataset. This means, based on MLM (or contrastive learning for the sentence similarity models), that the model already “agreed” that the co-cited papers were more semantically similar than other pairs that are not co-cited. This is the evidence behind co-citations as a measurement for similarity, but it also opens the door for future work. We believe it would be fruitful to explore our scheme on a paired dataset where the “natural” semantic similarity after MLM was less than 1.0 but is paired by some similarity heuristic. Many molecular sequences, like proteins, share this property, where pretrained transformers often lack true semantic embeddings after MLM alone^63,64^.

Of note, routing each example to a single expert based on the domain of the input means that the active parameters for the model are exactly equivalent to the model before MoE extension. In other words, the forward pass FLOPs are exactly equivalent to the original pretrained model. For homogeneous (all examples from one domain) inference batches, the throughput of the MoE extended version of the model vs. the original will be exactly the same as long as there is enough VRAM to store the additional inactive weights. Even without the necessary VRAM, portions of the model could be stored in the CPU memory when doing inference for a certain task, and then they could be switched for another subsequent task. Unfortunately, with heterogeneous batches during inference or training, the forward and backward pass will be slower based on how many experts need to be called. While requiring the same amount of FLOPs, they are not perfectly parallelized in the forward pass using naive implementations of switch versions for MoE. Issues like this may be alleviated in the future by efficient MoE parallelization efforts like DeepSeek’s DeepEP project^4,65^. For the backward pass, it is necessarily slower as more weights and gradients are involved. Even with naive implementations, we conclude from the strong performance across all five diverse evaluated domains that compute may be better spent on an *N* expert-wide MoE network than fine-tuning *N* equivalent networks, especially when considering domain overlap and the possibility of merging and LoRA methodologies.

We believe that there are many potential applications of the MoE extension framework coupled with contrastive and/or other fine-tuning methods. One compelling avenue is named entity recognition^66^. Because experts are only routed examples from a specific user-defined domain, an expert may produce informative hidden states surrounding niche and nuanced terms. We suspect the token-wise embeddings from intermediate experts or from the last hidden state may present strong correlations with downstream NER tasks due to specific domain embedding structures. Once pooled, the fixed-length vector embeddings may offer a useful platform for retrieval tasks, including Retrieval Augmented Generation (RAG). Pre-embedded datasets will be much more separable for intra-domain searches from prompted LLM systems, capable of returning closely related content based on domain-specific context. Even inter-domain searches may be ideally separable if the system was trained like MoE$_{all}$, with an MNR-like loss on multiple domains at once. One application that could benefit from separable inter-domain embeddings would be clinical or medical notes, where token-wise or pooled embeddings are fed to experts trained on other notes from a particular medical specialty or practice.

Another exciting application lies in mechanistic interpretability. We chose sentence-wise routing over other reasonable MoE routing schemas to keep expert weights completely separate for distinct domains. We believe this type of scheme is an ideal playground for mechanistic interpretability, examining nuanced and niche concepts learned for specific domains. There are many relevant questions here: How are the expert weights structured? Do sparse autoencoders reveal distinct neuron activations for niche concepts consistent across domains and experts^67^? Do the expert MLPs act like specified Hopfield networks^68,69^? It may also be possible to conduct weight merging or ensembling to increase performance and reduce VRAM costs^70^. Such research may also want to augment the attention layers in a domain-specific way. To accommodate this, we have included code to apply Low Rank Adaptation (LoRA) to attention layers, allowing for researchers to train domain-specific adapters alongside the MoE extended MLPs^71^.

Overall, our use of co-citation networks enables rapid and efficient dataset compilation for training transformers on niche scientific domains. The fine-tuning of base BERT models through contrastive learning with an MNR-inspired loss significantly improves sentence similarity capabilities. The MoE approach further expands these capabilities, suggesting the feasibility of a universal model for text classification and vector embeddings across various domains through MoE seeding and enforced routing. Given efficient inference considerations, one could embed large datasets such as the entire Semantic Scholar database without any additional overhead for using a MoE model. Using the building blocks of our approach, effective BERT models with specialized knowledge across multiple fields, vocabularies, or tasks can be developed.

## Supplementary Information

Supplementary Information.

## Data Availability

Links to all training data, model weights, and code can be found at Github [Gleghorn Lab, Mixture of Experts Sentence Similarity].
