# Supplementary material for: Contrastive learning and mixture of experts enables precise vector embeddings in biological databases
Source: Sci Rep. 2025 Apr 29;15:14953. doi: 10.1038/s41598-025-98185-8 (PMC12041245; doi:10.1038/s41598-025-98185-8)
Supplement: Supplementary file 1 — Supplementary Information. [file 41598_2025_98185_MOESM1_ESM.pdf]

Contrastive Learning and Mixture of Experts Enables Precise Vector Embeddings

Logan Hallee[1], Rohan Kapur[2], Arjun Patel[3], Jason P. Gleghorn[4*], and Bohdan Khomtchouk[5*]

[1]Center for Bioinformatics and Computational Biology, University of Delaware
[2]Department of Physics, University of Chicago
[3]The College of the University of Chicago
[4]Department of Biomedical Engineering, University of Delaware
[5]Department of BioHealth Informatics, Luddy School of Informatics, Computing, and Engineering, Indiana University
[*]Correspondence to gleghorn@udel.edu and bokhomt@iu.edu

Supplementary Information

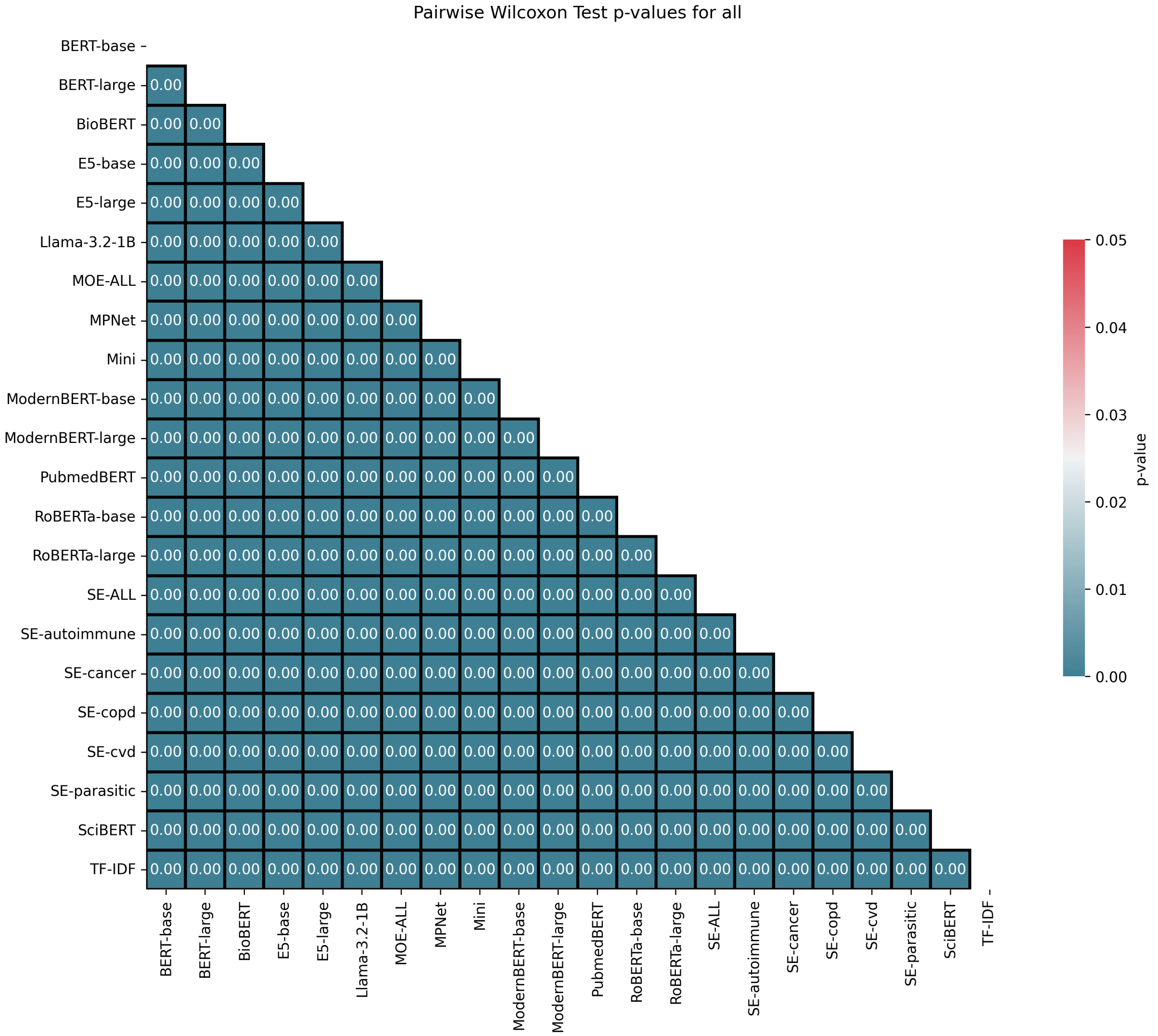

Figure 1. p-values plotted for pairwise Wilcoxon tests for all evaluated models on all the evaluation data.

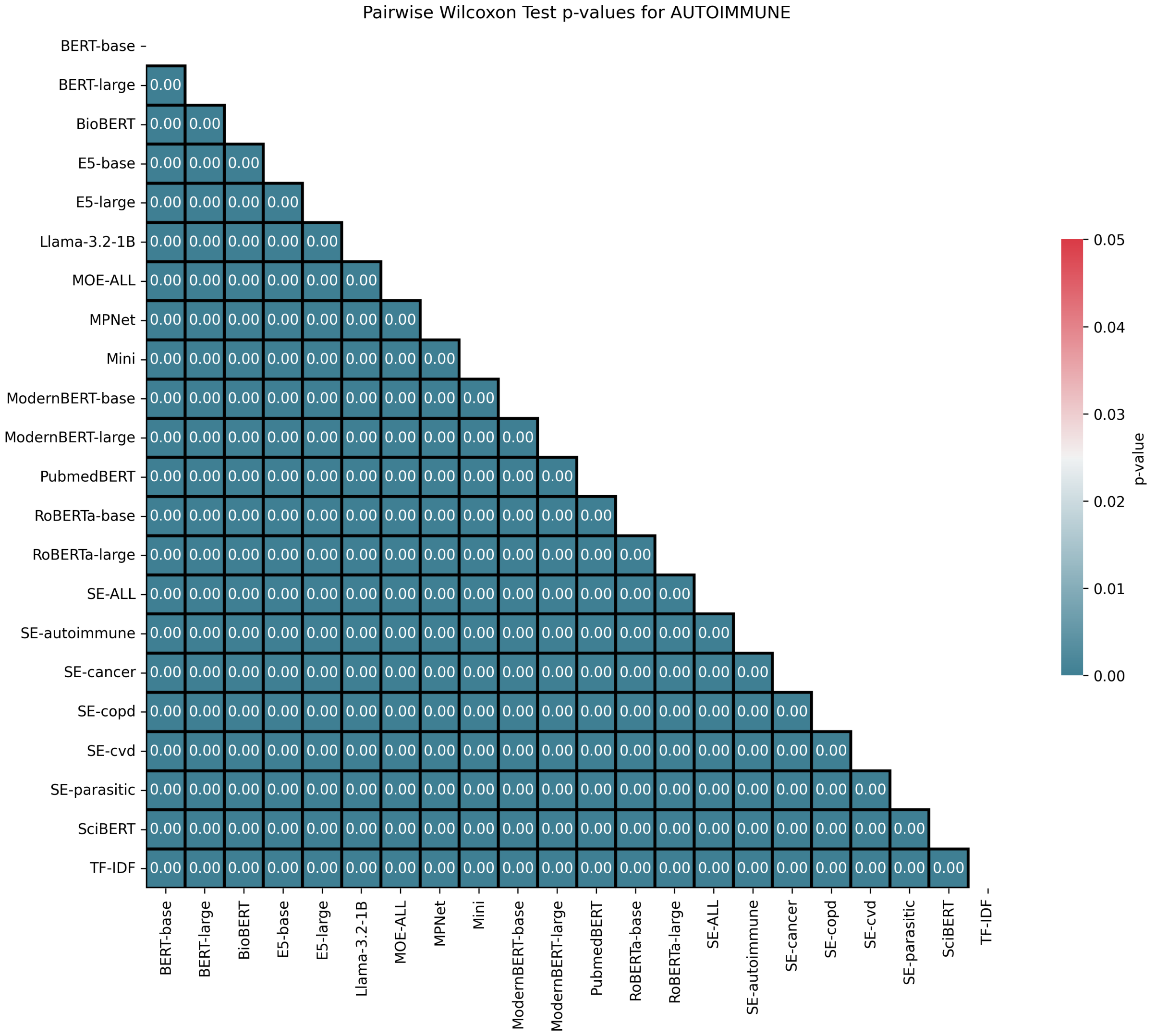

Figure 2. p-values plotted for pairwise Wilcoxon tests for all evaluated models on the autoimmune evaluation data.

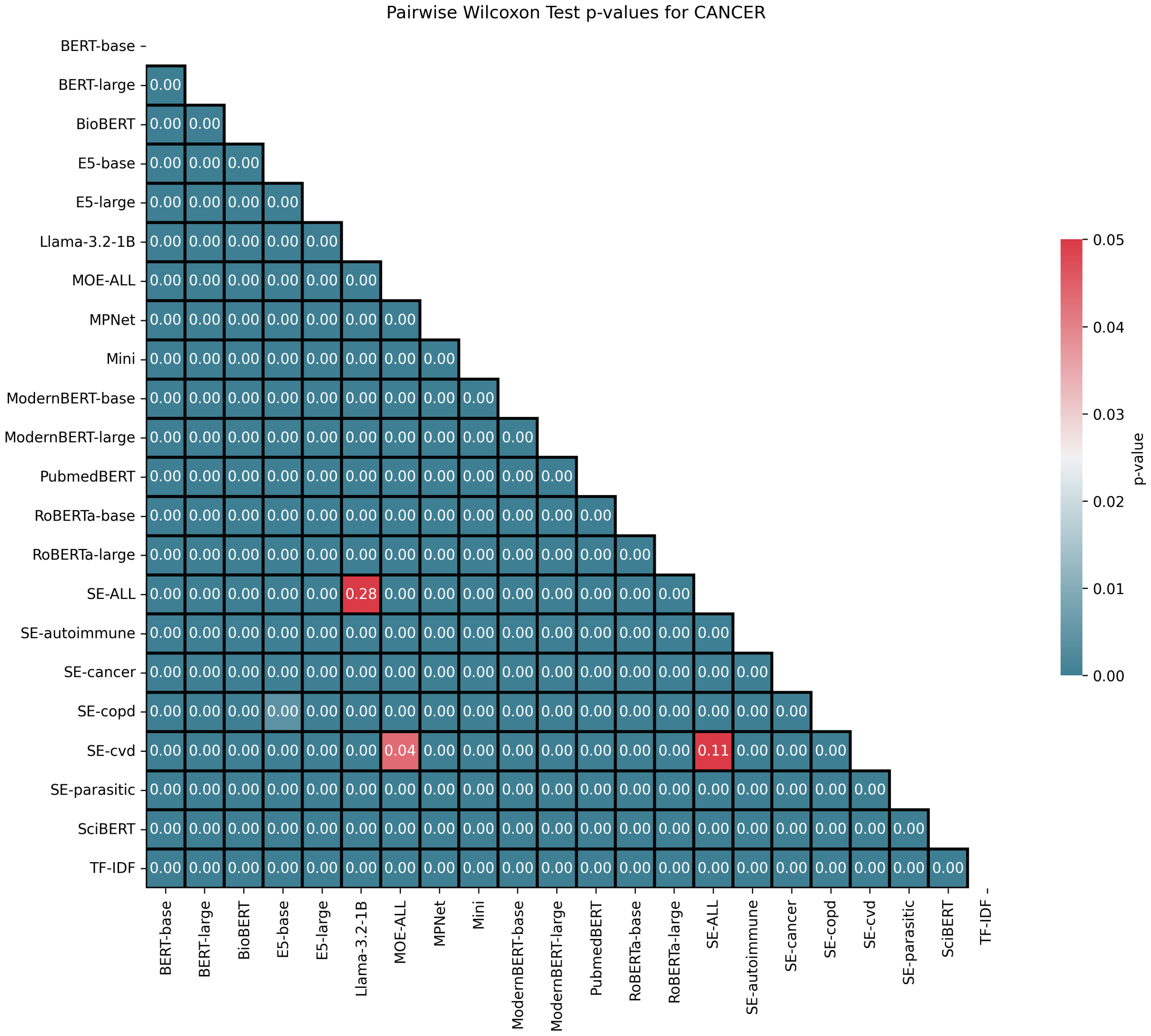

Figure 3. p-values plotted for pairwise Wilcoxon tests for all evaluated models on the skin cancer evaluation data.

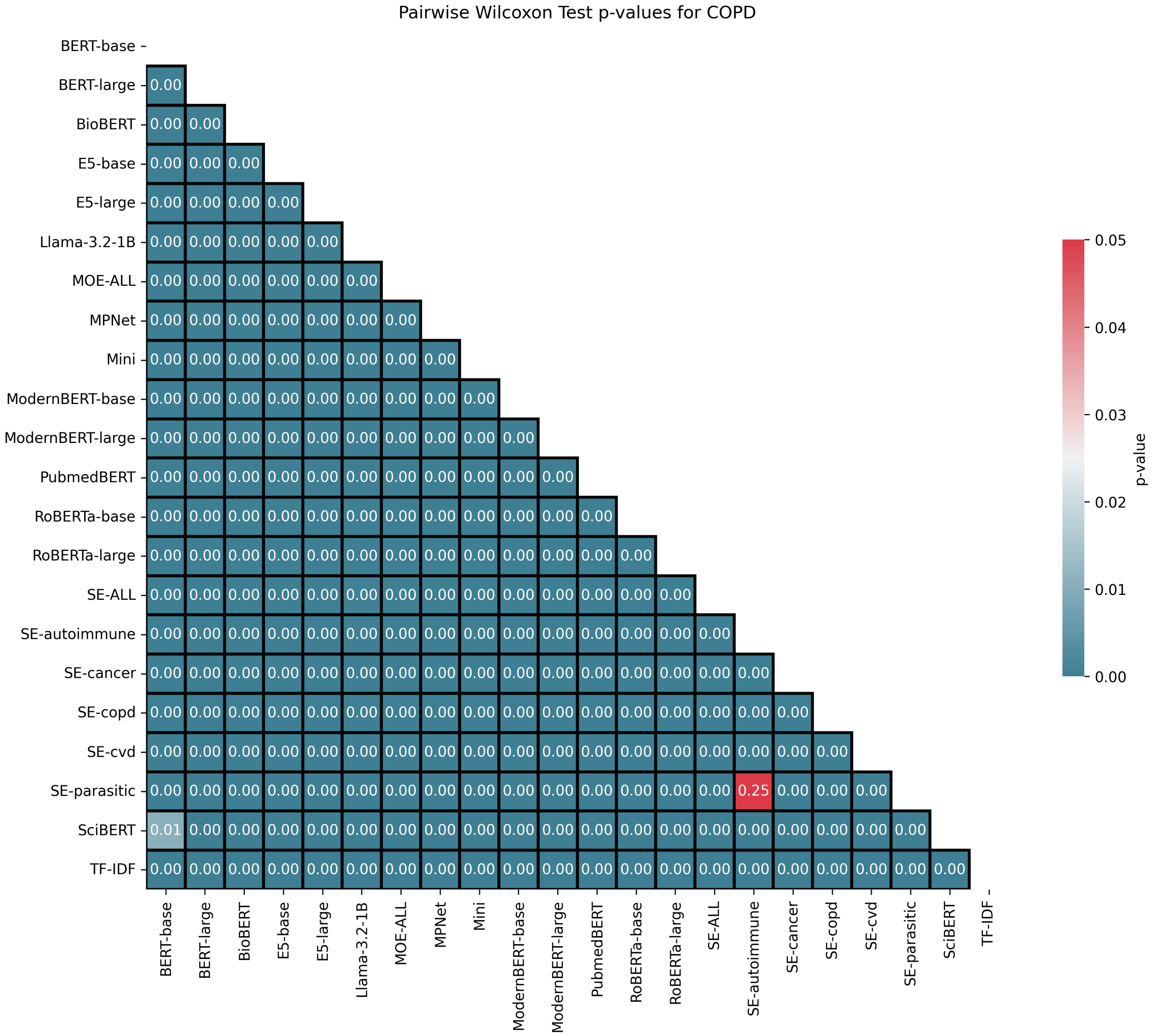

Figure 4. p-values plotted for pairwise Wilcoxon tests for all evaluated models on the COPD evaluation data.

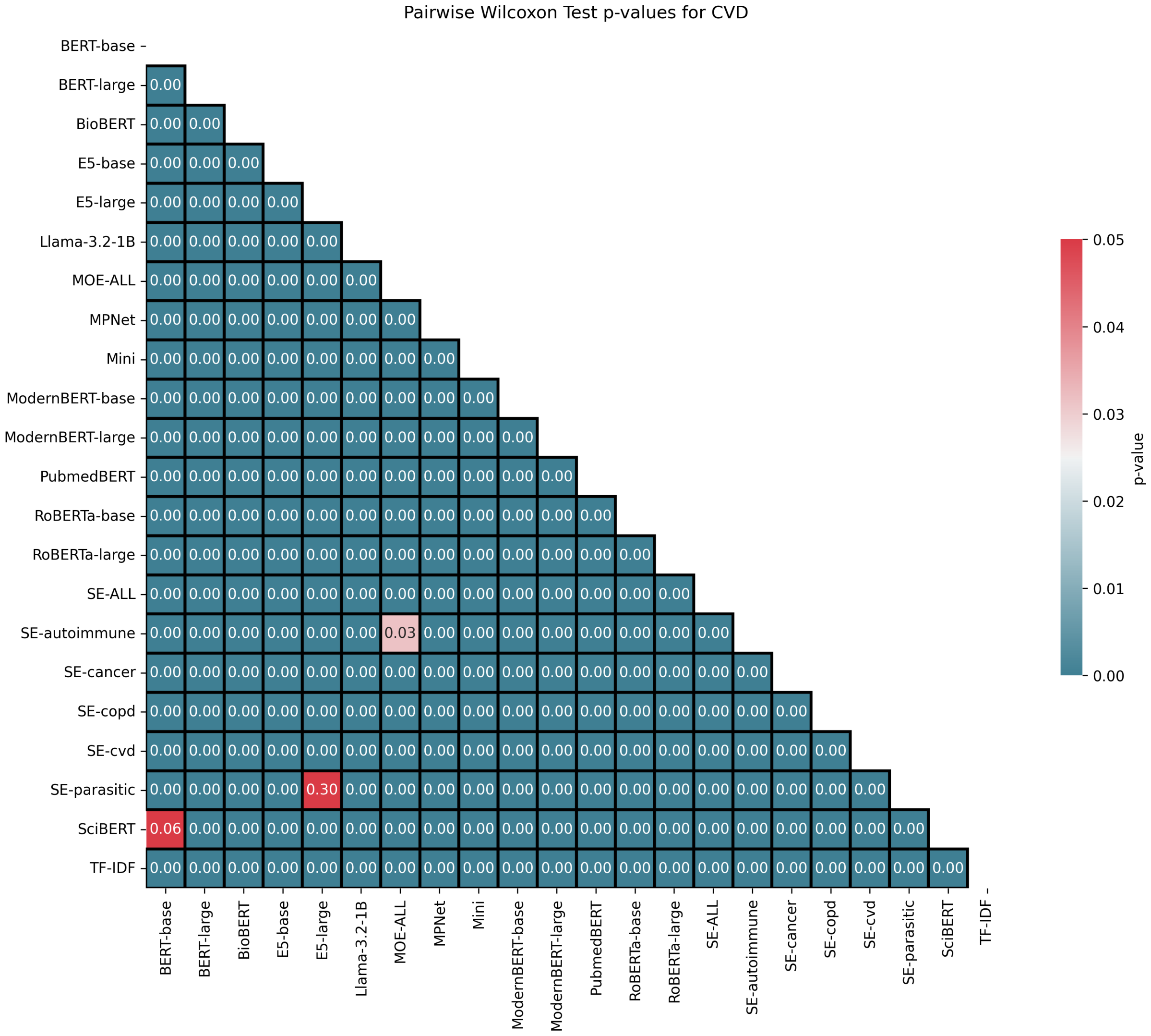

Figure 5. *p*-values plotted for pairwise Wilcoxon tests for all evaluated models on the CVD evaluation data.

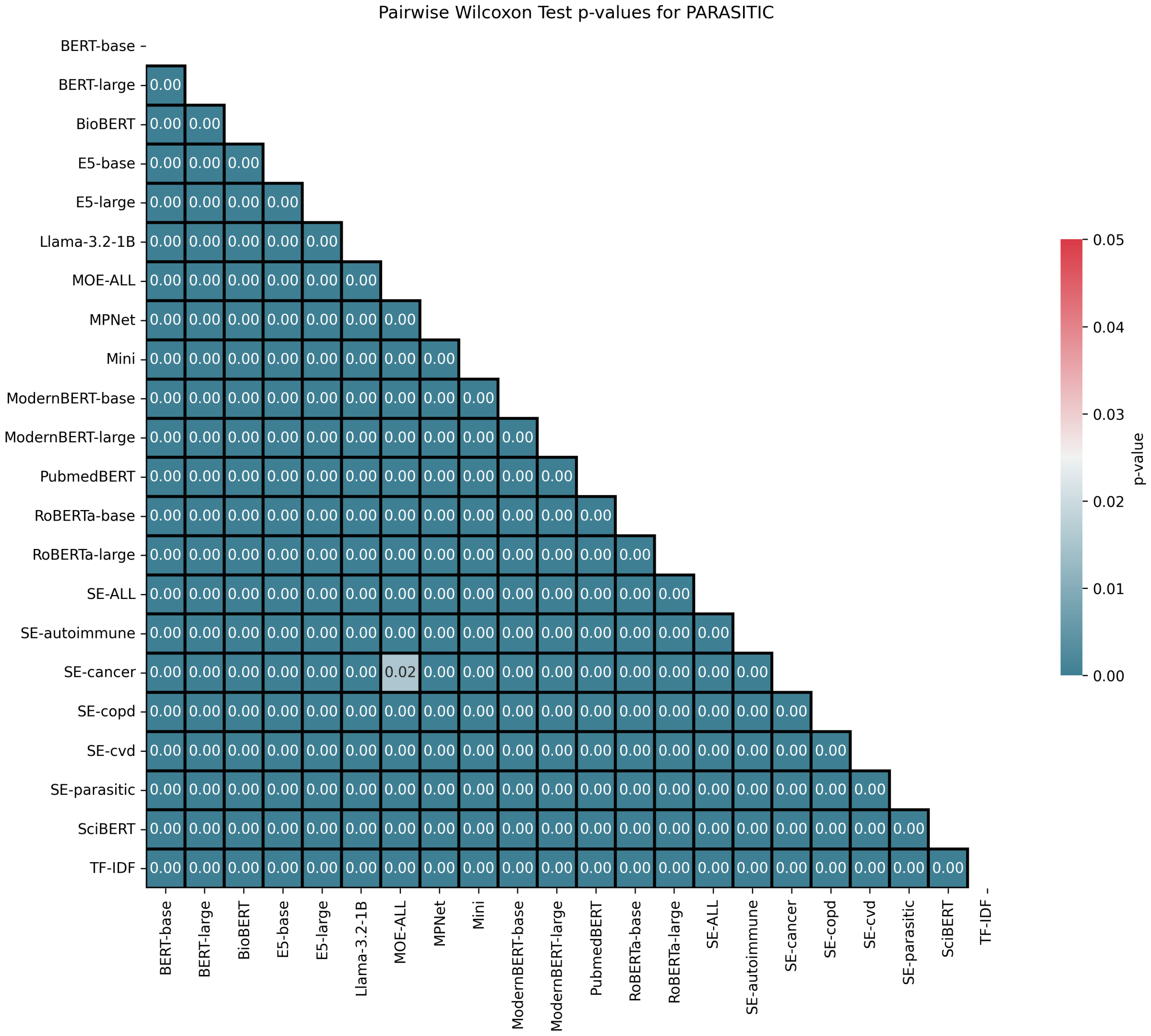

Figure 6. *p*-values plotted for pairwise Wilcoxon tests for all evaluated models on the parasitic evaluation data.
